# Draft Genome Sequences of Two Ulvan-Degrading Isolates, Strains LTR and LOR, That Belong to the *Alteromonas* Genus

**DOI:** 10.1128/genomeA.01081-14

**Published:** 2014-10-23

**Authors:** Moran Kopel, William Helbert, Bernard Henrissat, Tirza Doniger, Ehud Banin

**Affiliations:** a Institute for Nanotechnology and Advanced Materials, Bar-Ilan University, Ramat Gan, Israel; b Mina and Everard Goodman Faculty of Life Sciences, Bar-Ilan University, Ramat Gan, Israel; c Centre de Recherches sur les Macromolécules Végétales, UPR-CNRS 5301, Université Joseph Fourier and Institut de Chimie Moléculaire de Grenoble, ICMG, FR-CNRS 2607, Grenoble, France; d Architecture et Fonction des Macromolécules Biologiques, Centre National de la Recherche Scientiﬁque and Aix-Marseille University, Marseille, France

## Abstract

Here, we report the draft genome sequence of two ulvan-degrading *Alteromonas* spp. isolated from the feces of the sea slug, *Aplysia*. These sequenced genomes display a unique ulvan degradation machinery compared with ulvanolytic enzymes previously identified in *Nonlabens ulvanivorans*.

## GENOME ANNOUNCEMENT

The green seaweed *Ulva* sp. is distributed worldwide and represents an important and underexploited biomass. The consumption of this algae as sea lettuce (1) and its “green tide” proliferation events (2) have stimulated interest toward the study of the algae’s main constituents, and more specifically its major cell-wall polysaccharide, ulvan (3). This water-soluble polysaccharide, essentially composed of l-rhamnose, d-glucuronic acid, l-iduronic acid, d-xylose, and sulfate, frequently appears as a repeat of the disaccharide ulvanobiuronic acid 3-sulfate (3). Ulvan has been found to present attractive physicochemical and biological properties for the food/feed, agriculture, pharmaceutical, and biomaterials industries (3). However, despite the growing interest in ulvan, very little is known about its enzymatic degradation and utilization. To our knowledge, *Nonlabens ulvanivorans*, is the only described bacterium known to break down ulvan (4–6). Two ulvan-degrading enzymes were isolated from this bacterium and characterized: the ulvan lyase and its accessory unsaturated β-glucuronyl hydrolase (7, 8).

*Alteromonas* sp. strains LTR and LOR were isolated by Collen from the feces of the sea slug *Aplysia punctata* feeding on *Ulva*, as described previously for *N. ulvanovorans* isolation (4). According to 16S rRNA gene-sequence analysis, they were affiliated with the genus *Alteromonas* and have 99% sequence identity to each other. *De novo* sequencing of the two ulvan-degrading *Alteromonas* spp. was conducted as described previously (6). Whole-genome sequencing was performed using Illumina HiSeq 2000 in 100-bp paired-end (X2) sequencing and an average coverage of approximately 1,933×. Reads were assembled using the *de-novo* assemblers AbySS (9) and Velvet (10) into 70 (*N*_50_, 217,514 bp) and 76 contigs and scaffolds (*N*_50_, 217,514 bp) for LTR and LOR strains, respectively. Both genomes’ total length are approximately 4.41 Mb with a mean GC content of 44.2%. Contig annotation was conducted using the RAST (Rapid Annotations using Subsystems Technology) server (11). The final draft of strains LTR and LOR contain, respectively, 3,969 or 3,971 coding sequences (out of which 2,535 or 2,534 possess annotated functions and 1,434 or 1,437 are hypothetical proteins), 64 tRNA genes, and 1–2 rRNA operons were found in both strains. The number of rRNA operons is consistent with the number of rRNA copies estimated by the Ribosomal RNA Database (rrnDB) for the family *Alteromonadaceae* (between 2 and 5 copies) (12). A plasmid-partitioning gene, *parA*, was detected in both drafts, which suggests the occurrence of a plasmid.

These draft genome sequences of the two ulvan-degrading *Alteromonas* spp. expand our knowledge on the bacterial mechanism that supports ulvan and *Ulvales* biomass utilization. Although both genomes lack the sequence of previously described ulvan-degrading enzymes found in the *N. ulvanivorans* genome, their ability to degrade ulvan implies the presence of a different set of enzymes, which await characterization.

### Nucleotide sequence accession numbers.

This whole-genome shotgun project has been deposited at DDBJ/EMBL/GenBank under the accession numbers JQHG00000000 and JQFW00000000 for LTR and LOR, respectively. The versions described in this paper are versions JQHG01000000 and JQFW01000000.
